# The need for a practitioners' social media code of conduct in Iran

**DOI:** 10.18502/jmehm.v12i16.2015

**Published:** 2019-12-01

**Authors:** Ahmad Sofi-Mahmudi

**Affiliations:** *Researcher, Non-Communicable Diseases Research Center, Endocrinology and Metabolism Research Institute, Tehran University of Medical Sciences, Tehran, Iran. *

There is a need for a social media code of conduct for dental (or in general, medical) practitioners in Iran. With the growing use of the internet and social networks in Iran, many dentists use social media, mainly Instagram, as an advertisement tool. There are almost no restrictions on their advertisements; they not only expose their patients’ identities and therefore violate the principle of confidentiality, but also sometimes use inappropriate ways and styles, which may injure dentists’ reputation in the society (1). 

Another problem is the increasing number of fake accounts that belong to individuals who introduce themselves as dental/medical practitioners. This problem is more evident in Twitter, where a huge number of students and practitioners tend not to use their real names. Sometimes they make medical recommendations or use inappropriate words that may make other users cynical about the profession. Moreover, others can take advantage of this situation by claiming to be dental/medical practitioners, which has happened several times. 

In the latest dentistry curriculum in Iran, there are no instructions for use of social media in the “medical ethics” course. However, Tehran University of Medical Sciences has developed a guideline for professional behavior in cyberspace in August 2017. It is a good step, but we need an upstream and nationwide action. It is also advisable to adopt other countries guidelines as they may have more experience in this regard.

For example, the General Dental Council and the General Medical Council (GMC) of the UK, and the Australian Health Practitioner Regulation Agency have produced policies and guidelines regarding the use of social media (1). The UK's General Medical Councils “Doctors' Use of Social Media” (2) is a useful document, and its original and Persian translation are freely available on the web. This can help students and practitioners see that social media is not a part of their private life and they need to be careful while using social media.

## Notes:

**Citation to this article:**

Ahmad Sofi-Mahmudi A. The need for a practitioners' social media code of conduct in Iran J Med Ethics Hist Med. 2019; 12: 16
